# Predictors of symptomatic myelopathy in degenerative cervical spinal cord compression

**DOI:** 10.1002/brb3.797

**Published:** 2017-08-11

**Authors:** Zdenek Kadanka, Blanka Adamova, Milos Kerkovsky, Zdenek Kadanka, Ladislav Dusek, Barbora Jurova, Eva Vlckova, Josef Bednarik

**Affiliations:** ^1^ Department of Neurology University Hospital Brno Brno Czech Republic; ^2^ Applied Neurosciences Research Group Central European Institute of Technology Masaryk University Brno Brno Czech Republic; ^3^ Department of Radiology University Hospital Brno Brno Czech Republic; ^4^ Institute of Biostatistics and Analyses Faculty of Medicine Masaryk University Brno Brno Czech Republic

**Keywords:** cervical radiculopathy, degenerative cervical myelopathy, magnetic resonance imaging, nonmyelopathic degenerative cervical cord compression, predictive model

## Abstract

**Objectives:**

To update a previously established list of predictors for neurological cervical cord dysfunction in nonmyelopathic degenerative cervical cord compression (NMDCCC).

**Material and Methods:**

A prospective observational follow‐up study was performed in a cohort of 112 consecutive NMDCCC subjects (55 women and 57 men; median age 59 years, range 40–79 years), either asymptomatic (40 subjects) or presenting with cervical radiculopathy or cervical pain (72 subjects), who had completed a follow‐up of at least 2 years (median duration 3 years). Development of clinical signs of degenerative cervical myelopathy (DCM) as the main outcome was monitored and correlated with a large number of demographic, clinical, electrophysiological, and MRI parameters including diffusion tensor imaging characteristics (DTI) established at entry.

**Results:**

Clinical evidence of the first signs and symptoms of DCM were found in 15 patients (13.4%). Development of DCM was associated with several parameters, including the clinical (radiculopathy, prolonged gait and run‐time), electrophysiological (SEP, MEP and EMG signs of cervical cord dysfunction), and MRI (anteroposterior diameter of the cervical cord and cervical canal, cross‐sectional area, compression ratio, type of compression, T2 hyperintensity). DTI parameters showed no significant predictive power. Multivariate analysis showed that radiculopathy, cross‐sectional area (CSA) ≤ 70.1 mm^2^, and compression ratio (CR) ≤ 0.4 were the only independent significant predictors for progression into symptomatic myelopathy.

**Conclusions:**

In addition to previously described independent predictors of DCM development (radiculopathy and electrophysiological dysfunction of cervical cord), MRI parameters, namely CSA and CR, should also be considered as significant predictors for development of DCM.

## 1 INTRODUCTION

Degenerative cervical cord compression detected by imaging methods, mostly magnetic resonance imaging (MRI), is a prerequisite for the clinical diagnosis of degenerative cervical myelopathy (DCM). This overarching term is preferred to describe the various degenerative conditions of the cervical spine that cause myelopathy, including most frequent cervical spondylotic myelopathy, but also degenerative disc disease and ossification of the posterior longitudinal ligament and of the ligamentum flavum (Nouri, Tetreault, Singh, Karadimas, & Fehlings, 2015). There is a considerable body of current research related to various aspects of DCM, including prognostic factors (Tetreault, Karpova, & Fehlings, 2015; Tetreault, Nouri, Singh, Fawcett, & Fehlings, 2014). In recent years, studies have demonstrated that asymptomatic degenerative cervical cord compression detected on MRI (Boden et al., 1990; Matsumoto et al., 1998; Teresi et al., 1987) may be of a prevalence that exceeds that of symptomatic myelopathy (Bednarik et al., 2004, 2008; Bednařík et al., 1998; Kovalova et al., 2016; Wilson et al., 2013). Knowledge of the prevalence, as well as the frequency, of myelopathy development, and of risk factors influencing this progression, however, is sparse (Wilson et al., 2013). Such knowledge would be of crucial importance to the practical management of asymptomatic degenerative cervical cord compression, and bear upon the important issue of indications for preventive surgical decompression.

In other studies, we have established that the presence of symptomatic cervical radiculopathy and central conduction deficit in the cervical cord, disclosed by electrophysiological methods—somatosensory (SEP) and/or motor‐evoked potentials (MEP)—were independent predictors for the development of symptomatic DCM (Bednarik et al., 2004, 2008; Bednařík et al., 1998). These results tally, in part, with those of an international survey undertaken by the spine care community (Wilson et al., 2013) that identified the presence of radiculopathy together with MRI evidence of intramedullar T2 hyperintensity as important factors influencing the decision to perform preventive decompressive surgery in nonmyelopathic patients with degenerative cervical cord compression.

Our previous study (Bednarik et al., 2008), although extensive, had several limitations. Most importantly, the patients, although lacking any clear myelopathic symptoms and/or signs (i.e., “nonmyelopathic”), were in fact not completely asymptomatic, as our cohort was recruited from consecutive patients referred for radiculopathy and/or cervical axial pain. The term “asymptomatic” degenerative cervical cord compression should be reserved for completely asymptomatic cases, while nonmyelopathic subjects with or without signs/symptoms of radiculopathy or cervical pain should be referred to in terms of “nonmyelopathic degenerative cervical cord compression” (NMDCCC). As one of two alternative criteria for MRI‐detected cervical cord compression, we used compression ratio (CR) < 0.4 that might preclude less severe diffuse compression to be included into the study.

Further, spinal cord T2 hyperintensity is considered an important risk factor by the spine care community (Wilson et al., 2013), and diffusion tensor imaging (DTI) parameters have shown the capacity to differentiate cervical myelopathy patients not only from normal individuals (Chen et al., 2016; Guan et al., 2015; Lee et al., 2015) but also from nonmyelopathic cervical cord compression cases (Kerkovsky et al., 2012), and further to correlate with severity of myelopathy (Rajasekaran et al., 2014), the segments of the cervical cord involved (Suetomi et al., 2016), and to predict postsurgical outcome (Arima et al., 2015). A re‐evaluation of the predictive model describing the risk of progression of NMDCCC to symptomatic myelopathy (Bednarik et al., 2008) was thus indicated, in a sample also including completely asymptomatic subjects with less severe stages of degenerative cervical cord compression and with the use of DTI parameters to validate the previous model.

## 2 MATERIAL & METHODS

The sample size calculation, about 120 patients, was based on an anticipated frequency of DCM development of about 18% over the course of 3 years (derived from the previous study, Bednarik et al., 2008) and the number of evaluated predictors (20).

The study sample here consisted of a cohort of consecutive subjects who had been referred to the Department of Neurology between January 2012 and December 2013 with clinical signs and symptoms of cervical radiculopathy, moderate‐to‐severe chronic or intermittent axial cervical pain, and volunteers in whom MRI signs of degenerative cervical cord compression had previously been detected during an epidemiological study focusing on the prevalence of degenerative cervical cord compression in the population of the province of South Moravia (Kovalova et al., 2016). The inclusion of volunteers from the epidemiological study, complying with the criteria for the current study into prospective evaluation, had been planned beforehand.

All subjects in the study had to comply with the following inclusion criteria:

- MR signs of degenerative compression of the cervical spinal cord with or without concomitant change in signal intensity from the cervical cord on T2/T1 images (see “Imaging” below)
- Absence of any current myelopathic clinical signs and symptoms that could probably be attributed to cervical cord involvement, from the following list.

Symptoms:

- Gait disturbance
- Numb and/or clumsy hands
- Lhermitte's phenomenon
- Bilateral arm paresthesias
- Weakness of lower or upper extremities
- Urinary urgency, frequency, or incontinence.

Signs:

- Corticospinal tract signs:
  ○ Hyperreflexia/clonus
  ○ Spasticity
  ○ Pyramidal signs (Babinski's or Hoffman's sign)
  ○ spastic paresis of any of the extremities (most frequently lower spastic paraparesis)
- Flaccid paresis of one or two upper extremities in the plurisegmental distribution
- Atrophy of hand muscles
- Sensory involvement in various distributions in upper or lower extremities (always plurisegmental)
- Gait ataxia with positive Romberg sign.

Originally, 137 NMDCCC subjects were included into the prospective evaluation. Twenty‐five subjects were lost during follow‐up and the follow‐up of at least 2 years was completed by a group of 112 subjects (55 women and 57 men; median age 59 years, range 34–79 years): 72 subjects had nonmyelopathic signs or symptoms probably related to degenerative changes of the cervical spine (namely axial pain and/or symptoms or signs of upper extremity monoradiculopathy), while 40 subjects were completely asymptomatic. The whole study cohort was a completely new sample, and none of these subjects had been included in a previously published prospective study on this topic (Bednarik et al., 2004).

Ethical approval for the study was granted by the Ethical Committee of the University Hospital, Brno.

### 2.1 Clinical evaluation

A detailed clinical examination was carried out at the beginning of the study and every 6 months thereafter. Patients were instructed about possible signs and symptoms that might indicate newly developed DCM and encouraged to arrange a consultation with a neurologist from the study group if they suspected a progression to myelopathy. The minimum follow‐up period was 24 months (median 30 months; range 24–48 years).

A standardized, timed 10‐m walk and run (as quickly as possible) was evaluated, in terms of time taken and number of steps required.

The primary end‐point of the study was the detection of development of symptomatic DCM based on the occurrence of at least one symptom and one sign (from the list used as exclusionary criteria—see above), which were probably attributed to degenerative cervical cord compression, were not present at the beginning of the follow‐up and had no other topical or etiological explanation.

Clinical evaluation focused on the determination of development of symptomatic myelopathy (as primary outcome) was performed by neurology specialists experienced in the diagnosis and practical management of myelopathic cases (ZK, ZKJ, MN) and the final decision on meeting the outcome, that is, development of symptomatic DCM, was approved by ZK, a senior neurologist with a long‐term experience in clinical studies on cervical myelopathy.

### 2.2 Imaging

Plain anteroposterior, oblique, and lateral radiograms were obtained in all patients. Their Torg–Pavlov ratio (TPR) at C5 level was calculated from lateral radiograms as the anteroposterior diameter of the spinal canal divided by the anteroposterior diameter of the vertebral body. All subjects underwent MRI examination of the cervical spine on a 1.5 T MR device with a 16‐channel head and neck coil. The standardized imaging protocol included conventional pulse sequences in sagittal‐T1, T2 and short‐tau inversion recovery (STIR) and axial planes (gradient‐echo T2) for the purpose of morphological evaluation and a DTI sequence in the axial plane coherently covering five segments of the cervical spine from levels C2/3 to C6/7. The DTI scans were acquired at a slice thickness of 4 mm, with the same geometry settings as those employed for the axial T2 images. The clinical status of patients/volunteers was blinded to the neuroradiologists who examined the cervical spine MRIs. The MRI of every subject was evaluated by two neuroradiologists, who agreed on the assessment of the compression in the majority of cases. Where disagreement existed—seldom—the final decision was based on a cooperative decision.

The imaging criterion for cervical cord compression was defined as a change in spinal cord contour or shape at the level of an intervertebral disc on axial or sagittal MRI scan compared to that at midpoint level of neighboring vertebrae.

**Figure 1:**
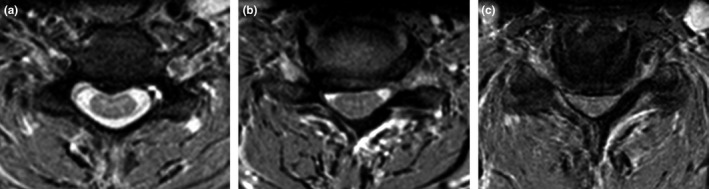
(a) Example of the “impingement” type of spondylotic cervical cord compression (type I): focal concave anterior defect of spinal cord contour and with preserved subarachnoid space. (b) Example of a flat compression with partially preserved subarachnoid space (type IIa). (c) Example of a flat compression with lost subarachnoid space (type IIb)
- Flat or circular compression with partially preserved subarachnoid space—type IIa (Figure 1b)—or with lost subarachnoid space—type IIb (Figure 1c).

Spinal cord compression was further graded as:

- Impingement, that is focal concave, usually anterior, defect of spinal cord contour and with preservation of a major part of subarachnoid space outside of the compression—type I (Figure 1a)

The following conventional MRI parameters were also measured: Cross‐sectional spinal cord area (CSA), anteroposterior (AP) and laterolateral (LL) spinal cord diameter, compression ratio considered in terms of anteroposterior/laterolateral spinal cord diameter (CR) (Arima et al., 2015; Wilson et al., 2013), circumference of spinal cord (CSC), and anteroposterior diameter of cervical canal (APo). These measurements were taken at the level of maximum spinal cord compression (MCL) identified as maximum reduction of AP spinal canal diameter in comparison with other segments. In patients with multisegmental involvement and a similar degree of spinal canal stenosis, the level with the smallest spinal cord area was chosen. The presence of T2 hyperintensity was also noted.

FiberTrak, Extended MR WorkSpace (release 2.6.3.5, Philips Medical Systems) was used for DTI data analysis. Diffusion data were processed and fractional anisotropy (FA) and apparent diffusion coefficient (ADC) values calculated. Measurements were subjected to region‐of‐interest (ROI) analysis by placing the ROIs at the level of intervertebral disks over the entire spinal cord area depicted on the axial images of isotropic diffusion. Mean FA and ADC values of the spinal cord cross‐sections were recorded at maximum compression level (MCL) in all NMDCCC subjects. FA (ADC) ratios were calculated as FA (ADC) at MCL levels divided by FA (ADC) at C2/3 level.

### 2.3 Electrophysiological evaluation

Short‐latency SEPs from the median and the tibial nerves were elicited at the beginning of the study by electrical stimulation of mixed nerves at the wrist and the ankle. Similarly, MEPs were elicited by means of transcranial and root magnetic stimulation and recorded from abductor digiti minimi and abductor hallucis muscles on both sides. Details on the methodology of electrophysiological examination and evaluation of results with definition of central conduction abnormality attributable to possible cervical spinal cord lesion are described in previous publications (Bednarik et al., 2004, 2008; Bednařík et al., 1998).

Motor and sensory conduction studies were performed on six motor nerves (median, ulnar, and tibial nerves bilaterally) and four sensory (ulnar and sural nerves bilaterally) using conventional techniques. Needle EMG from four muscles (deltoid, biceps brachii, triceps brachii, and first dorsal interosseous) was performed bilaterally with assessment of spontaneous activity, motor unit potential parameters, and interference patterns. EMG signs of acute motor axonal neuropathy in one myotome (C5–Th1) corresponding with radicular signs and symptoms were classified as radicular. EMG signs of acute, subacute, or chronic motor axonal neuropathy, established in more than one myotome (C5–Th1) unilaterally or bilaterally, were classified as signs of anterior horn cell lesion resulting from degenerative cervical myelopathy.

The following variables were recorded at the entry examination and their association with the predefined end‐points (i.e., development of clinically symptomatic DCM and time taken for it) were analyzed.

### 2.4 Demographic and clinical data

- Age
- Sex
- Baseline clinical status:
  ○ Presence of clinical symptoms and signs of cervical radiculopathy (with corresponding CT and/or MR findings and, in the case of motor deficit with corresponding EMG findings, of motor axonal neuropathy in one myotome)
  ○ Cervical pain
  ○ Randomly recruited asymptomatic subjects
- 10‐m timed walk (time and number of steps)
- 10‐m timed run (time and number of steps).

### 2.5 Electrophysiological data

- Abnormal SEP interpreted as lesion in either segmental dorsal horn or dorsal column
- Abnormal MEP interpreted as lesion of corticospinal tract
- Abnormal EMG signs of plurisegmental anterior horn cell lesion.

### 2.6 Imaging data

- TPR
- AP, LL, CR, CSC, CSA
- FA and ADC at MCL level
- FA and ADC ratios
- T2 hyperintensity
- Type of MRI‐detected cervical cord compression
- Maximum stenotic level and number of stenotic levels

### 2.7 Statistical analysis

Standard univariate statistical techniques were used to test differences between the chosen subgroups of patients and association between the parameters examined: Fisher's exact test for binary outcomes (or its extension—Fisher–Freeman–Halton exact test for contingency tables larger than 2 × 2) and Mann–Whitney *U* test for continuous variables.

The power of parameters to discriminate between NMDCCC subjects who developed symptomatic DCM and those who remained asymptomatic was evaluated by receiver operating curve (ROC) analysis and expressed as area under curve (AUC), with sensitivity and specificity based on established cut‐off values. The power of parameters to predict development of DCM was calculated using univariate logistic regression. All continuous parameters were also coded as binary predictors on the basis of cut‐off points defined in ROC analysis.

Finally, multivariate model—adjusted logistic regression—was used to seek independent predictors for the development of symptomatic DCM. The variables were selected using a forward step‐wise selection algorithm.

## 3 RESULTS

Clinical evidence of the first signs and symptoms of DCM within the entire follow‐up period was found in 15 patients (13.4%): the DCM+ subgroup. DCM developed in seven cases (6.3%) during the first 12 months of the follow‐up period. The frequency of myelopathic symptoms and signs in our cohort are summarized in Table 1. Gait disturbance was the most frequent symptom, followed by numb or clumsy hands, while corticospinal tract signs represented dominant initial clinical presentation on neurological examination.

**Table 1:** Frequency of myelopathic symptoms and signs in 15 patients with newly developed DCM

	Frequency (no of patients)
Symptoms	
Gait disturbance	9
Numb and/or clumsy hands	7
Weakness of lower extremity	3
Bilateral arm paresthesias	2
Lhermitte's phenomenon	1
Signs	
Hyperreflexia/clonus	5
Pyramidal signs (Babinski's or Hoffman's sign)	4
Sensory involvement (plurisegmental)	3
Gate ataxia	3
Flaccid paresis of upper extremity (plurisegmental)	3
Spastic paresis of lower extremity, spastic gate	2

Baseline characteristics for the development of symptomatic cervical myelopathy are summarized in Table 2. Demographic factors (age, sex), maximum compression level, Torg–Pavlov ratio and DTI parameters showed no difference in distribution between DCM+ subgroup and those who did not develop symptomatic DCM (DCM− subgroup). Several clinical (baseline clinical symptoms or signs, parameters of gait and run), electrophysiological (SEP, MEP, EMG), and imaging parameters (type of compression, T2 hyperintensity, APo, AP, CSA, CR), however, displayed differences between DCM+ and DCM− subgroups.

**Table 2:** Baseline characteristics in relation to the development of symptomatic cervical myelopathy

Parameter^a^	Total (*n* = 112)	DCM+ (*n* = 15)	DCM− (*n* = 97)	*p* b
Sex (male)	57 (50.9%)	8 (53.3%)	49 (50.5%)	.999
Age	59.0 (34.0; 79.0)	58.0 (42.0; 77.0)	59.0 (34.0; 79.0)	.898
Baseline clinical status				
Asymptomatic	40 (35.7%)	2 (13.3%)	38 (39.2%)	**.015**
Cervical pain	50 (44.6%)	6 (40.0%)	44 (45.4%)	
Radiculopathy	22 (19.6%)	7 (46.7%)	15 (15.5%)	
Gait: time (s)	6.0 (3.8; 19.7)	8.8 (4.0; 19.7)	6.0 (3.8; 16.0)	**.015**
Gait: steps	13.0 (6.0; 29.0)	18.0 (10.0; 29.0)	13.0 (6.0; 21.0)	**.002**
Run: time (s)	4.0 (2.2; 13.0)	5.1 (3.0; 13.0)	4.0 (2.2; 8.0)	**.003**
Run: steps	11.0 (7.0; 23.0)	12.0 (8.0; 23.0)	11.0 (7.0; 19.0)	**.143**
EMG signs of myelopathy	7 (6.3%)	3 (20.0%)	4 (4.1%)	**.049**
Abnormal MEP	10 (8.9%)	5 (33.3%)	5 (5.2%)	**.004**
Abnormal SEP	17 (15.2%)	6 (40.0%)	11 (11.3%)	**.011**
Torg–Pavlov ratio	0.9 (0.5; 1.5)	0.9 (0.6; 1.2)	0.9 (0.5; 1.5)	.187
Maximum compression level				
C3/4	15 (13.4%)	2 (13.3%)	13 (13.4%)	.668
C4/5	25 (22.3%)	4 (26.7%)	21 (21.6%)	
C5/6	61 (54.5%)	9 (60.0%)	52 (53.6%)	
C6/7	11 (9.8%)	0 (0.0%)	11 (11.3%)	
Type of compression				
I	42 (37.5%)	1 (6.7%)	41 (42.3%)	**.005**
IIA	47 (42.0%)	7 (46.7%)	40 (41.2%)	
IIB	23 (20.5%)	7 (46.7%)	16 (16.5%)	
APo (mm)	8.0 (4.7; 12.6)	7.5 (5.1; 9.8)	8.3 (4.7; 12.6)	**.015**
AP (mm)	6.7 (4.7; 8.7)	6.1 (4.7; 7.5)	6.7 (4.8; 8.7)	**.015**
LL (mm)	14.6 (12.3; 17.3)	14.6 (13.0; 15.8)	14.6 (12.3; 17.3)	.966
SCC (mm)	36.4 (31.0; 42.9)	35.7 (33.4; 39.0)	36.5 (31.0; 42.9)	.356
CSA (mm^2^)	78.7 (53.0; 103.7)	67.1 (53.0; 88.4)	79.4 (54.4; 103.7)	**.001**
CR	0.5 (0.3; 0.6)	0.4 (0.3; 0.5)	0.5 (0.3; 0.6)	**.004**
T2 hyperintensity	11 (9.8%)	5 (33.3%)	6 (6.2%)	**.006**
FA MCL	0.5 (0.3; 0.7)	0.5 (0.4; 0.6)	0.5 (0.3; 0.7)	.620
ADC MCL	1.2 (0.6; 1.6)	1.2 (1.0; 1.4)	1.1 (0.6; 1.6)	.093
FA ratio	0.9 (0.5; 1.6)	0.9 (0.6; 1.1)	0.9 (0.5; 1.6)	.513
ADC ratio	0.9 (0.6; 1.5)	0.9 (0.7; 1.1)	0.9 (0.6; 1.5)	.522

ADC, apparent diffusion coefficient; ADC ratio, ADC at MCL level/C2/3 level; AP, anteroposterior spinal cord diameter; APo, anteroposterior cervical canal diameter; CR, compression ratio; CSA, cross‐sectional spinal cord area; DCM, degenerative cervical myelopathy; EMG, electromyography; FA, fractional anisotropy; FA ratio, FA at MCL level/C2/3 level; LL, laterolateral spinal cord diameter; MCL, maximum compression level; MEP, motor‐evoked potentials; SCC, spinal cord circumference; SEP, somatosensory‐evoked potentials.

a Median (minimum–maximum) values were used for continuous variables; absolute and relative frequencies were used for categorical variables. Statistically significant differences are expressed in bold type (*p* < .05).

b Mann–Whitney *U* test was used for continuous variables; Fisher's exact test or Fisher–Freeman–Halton exact test was used for categorical variables.

Some of these parameters were able to discriminate significantly NMDCCC subjects who developed symptomatic DCM (*n* = 15) from those who remained asymptomatic (*n* = 97) (Table 3). Furthermore, the predictive value of parameters to forecast development of DCM using univariate logistic regression and Cox proportional hazard models was evaluated (Table 4). Among significant predictors were the presence of radiculopathy, quantitative gait and run parameters, electrophysiological signs of cervical cord dysfunction detected by SEP, MEP and EMG, and several radiological parameters: type IIB of MRI compression, APo, AP, CSA, CR, and the presence of T2 hyperintensity. DTI parameters showed no significant predictive power.

**Table 3:** Discrimination power of parameters to distinguish between NMDCCC subjects who developed symptomatic DCM (*n* = 15) and those that remained asymptomatic (*n* = 97)

Parameter	AUC (95% CI)^a^	*p*	Cut‐off	Sensitivity (%)	Specificity (%)
Sex (male)	0.514 (0.357; 0.672)	.861	—	53.3	49.5
Age	0.510 (0.358; 0.662)	.898	≤59.5	66.7	49.5
Gait: time (s)	0.696 (0.534; 0.858)	**.015**	≥7.35	80.0	66.0
Gait: steps	0.754 (0.601; 0.906)	**.002**	≥17.5	53.3	90.7
Run: time (s)	0.743 (0.584; 0.901)	**.004**	≥4.95	71.4	72.2
Run: steps	0.621 (0.457; 0.784)	.148	≥12.5	50.0	74.4
EMG signs of myelopathy	0.579 (0.410; 0.748)	.324	—	20.0	95.9
Abnormal MEP	0.641 (0.470; 0.812)	.080	—	33.3	94.8
Abnormal SEP	0.643 (0.476; 0.810)	.075	—	40.0	88.7
Torg–Pavlov ratio	0.606 (0.455; 0.757)	.188	≤0.925	73.3	51.5
APo (mm)	0.695 (0.553; 0.838)	**.015**	≤8.4	93.3	42.3
AP (mm)	0.694 (0.546; 0.842)	**.016**	≤5.75	46.7	89.7
LL (mm)	0.503 (0.346; 0.661)	.966	≤15.95	100.0	10.3
SCC (mm)	0.574 (0.431; 0.718)	.356	≤34.35	33.3	84.5
CSA (mm^2^)	0.760 (0.624; 0.897)	**.001**	≤70.1	66.7	82.5
CR	0.733 (0.588; 0.877)	**.004**	≤0.40	60.0	89.7
T2 hyperintensity	0.636 (0.466; 0.806)	.092	—	33.3	93.8
FA MCL	0.540 (0.407; 0.673)	.620	≤0.5975	93.3	24.7
ADC MCL	0.635 (0.522; 0.748)	.093	≥1.089	93.3	42.3
FA ratio	0.553 (0.424; 0.681)	.513	≤1.0205	93.3	30.9
ADC ratio	0.552 (0.401; 0.702)	.522	≥0.938	53.3	64.9

ADC, apparent diffusion coefficient; ADC ratio, ADC at MCL level/C2/3 level; AP, anteroposterior spinal cord diameter; APo, anteroposterior cervical canal diameter; CR, compression ratio; CSA, cross‐sectional spinal cord area; DCM, degenerative cervical myelopathy; EMG, electromyography; FA, fractional anisotropy; FA ratio, FA at MCL level/C2/3 level; LL, laterolateral spinal cord diameter; MCL, maximum compression level; MEP, motor‐evoked potentials; SCC, spinal cord circumference; NMDCCC, nonmyelopathic degenerative cervical cord compression; SEP, somatosensory‐evoked potentials.

a Area under the curve (95% CI) and its statistical significance, based on ROC analysis. Statistically significant discriminating powers are expressed in bold type (*p* < .05).

**Table 4:** Predictive power of parameters to distinguish between NMDCCC subjects who developed symptomatic DCM (*n* = 15) and those that remained asymptomatic (*n* = 97) using univariate analysis

Parameter	Univariate logistic regression models		Univariate Cox proportional hazard models	
	Odds ratio (95% CI)	*p*	Hazard ratio (95% CI)	*p*
Sex (male)	1.120 (0.377; 3.329)	.839	1.102 (0.400; 3.039)	.851
Age	1.004 (0.949; 1.063)	.888	1.005 (0.952; 1.061)	.858
≤59.5	1.959 (0.624; 6.156)	.250	1.791 (0.612; 5.243)	.287
Clinical status at entry				
Asymptomatic	ref.		ref.	
Cervical pain	2.591 (0.494; 13.601)	.260	2.353 (0.000; 0.000)	.296
Radiculopathy	8.867 (1.650; 47.635)	**.011**	6.177 (0.000; 0.000)	**.024**
Gait: time (s)	1.324 (1.112; 1.576)	**.002**	1.235 (1.099; 1.388)	**<.001**
≥7.35	7.758 (2.045; 29.422)	**.003**	6.425 (1.811; 22.796)	**.004**
Gait: steps	1.359 (1.145; 1.613)	**<.001**	1.253 (1.132; 1.388)	**<.001**
≥17.5	11.175 (3.284; 38.022)	**<.001**	7.610 (2.749; 21.067)	**<.001**
Run: time (s)	1.802 (1.249; 2.601)	**.002**	1.368 (1.161; 1.613)	**<.001**
≥4.95	5.760 (1.795; 18.484)	**.003**	4.625 (1.578; 13.553)	**.005**
Run: steps	1.206 (1.017; 1.430)	**.032**	1.154 (1.010; 1.318)	**.035**
≥12.5	2.815 (0.921; 8.603)	.069	2.515 (0.912; 6.939)	.075
EMG signs of myelopathy	5.812 (1.158; 29.171)	**.032**	4.084 (1.151; 14.491)	**.029**
Abnormal MEP	9.200 (2.267; 37.341)	**.002**	6.130 (2.084; 18.030)	**.001**
Abnormal SEP	5.212 (1.556; 17.456)	**.007**	4.114 (1.462; 11.571)	**.007**
Torg–Pavlov ratio	0.084 (0.002; 3.522)	.194	0.105 (0.003; 3.356)	.203
≤0.925	2.926 (0.871; 9.827)	.082	2.623 (0.834; 8.249)	.099
Maximum compression level				
C3/4	ref.		ref.	
C4/5	1.238 (0.198; 7.741)	.819	1.177 (0.215; 6.459)	.851
C5/6	1.125 (0.216; 5.848)	.889	1.049 (0.226; 4.870)	.952
C6/7	—		—	
Type of compression				
I	ref.		ref.	
IIA	7.175 (0.844; 60.989)	.071	6.363 (0.783; 51.715)	.083
IIB	17.937 (2.041; 157.650)	**.009**	14.520 (1.784; 118.149)	**.012**
APo (mm)	0.540 (0.338; 0.864)	**.010**	0.581 (0.390; 0.865)	**.008**
≤8.4	10.250 (1.296; 81.097)	**.027**	9.251 (1.216; 70.398)	**.032**
AP (mm)	0.398 (0.190; 0.835)	**.015**	0.450 (0.238; 0.852)	**.014**
≤5.75	7.612 (2.276; 25.456)	**.001**	5.683 (2.053; 15.730)	**.001**
LL (mm)	0.974 (0.564; 1.680)	.923	0.989 (0.595; 1.645)	.967
≤15.95	—		—	
SCC (mm)	0.912 (0.723; 1.150)	.436	0.928 (0.751; 1.147)	.491
≤34.35	2.733 (0.818; 9.133)	.102	2.310 (0.789; 6.766)	.127
CSA (mm^2^)	0.911 (0.859; 0.966)	**.002**	0.925 (0.882; 0.971)	**.002**
≤70.1	9.412 (2.851; 31.071)	**<.001**	7.002 (2.388; 20.529)	**<.001**
CR (0.1 increase)	0.157 (0.051; 0.481)	**.001**	0.217 (0.089; 0.529)	**.001**
≤0.40	13.050 (3.842; 44.329)	**<.001**	8.504 (3.018; 23.962)	**<.001**
T2 hyperintensity	7.583 (1.957; 29.387)	**.003**	5.105 (1.737; 15.000)	**.003**
FA MCL	0.280 (0.000; 320.502)	.723	0.369 (0.001; 254.941)	.765
≤0.5975	4.603 (0.575; 36.861)	.150	4.135 (0.543; 31.474)	.170
ADC MCL	8.197 (0.348; 193.260)	.192	6.195 (0.369; 104.003)	.205
≥1.089	10.250 (1.296; 81.097)	**.027**	9.038 (1.188; 68.753)	**.033**
FA ratio	0.334 (0.015; 7.428)	.488	0.392 (0.023; 6.715)	.518
≤1.0205	6.269 (0.788; 49.874)	.083	5.657 (0.744; 43.030)	.094
ADC ratio	2.555 (0.054; 119.886)	.633	2.547 (0.077; 84.577)	.601
≥0.938	2.118 (0.707; 6.341)	.180	2.031 (0.736; 5.606)	.171

ADC, apparent diffusion coefficient; ADC ratio, ADC at MCL level/C2/3 level; AP, anteroposterior spinal cord diameter; APo, anteroposterior cervical canal diameter; CR, compression ratio; CSA, cross‐sectional spinal cord area; DCM, degenerative cervical myelopathy; EMG, electromyography; FA, fractional anisotropy, FA ratio, FA at MCL level/C2/3 level; LL, laterolateral spinal cord diameter; MCL, maximum compression level; MEP, motor‐evoked potentials; NMDCCC, nonmyelopathic degenerative cervical cord compression; SCC, spinal cord circumference; SEP, somatosensory‐evoked potentials. All continuous parameters were also coded as binary predictors on the basis of cut‐off points defined in ROC analysis. Statistically significant predictive powers are expressed in bold type (*p* < .05).

Multivariate analysis using multivariate‐adjusted logistic regression model, however, disclosed radiculopathy, CSA ≤ 70.1 mm^2^, and CR ≤ 4.0 as being the only independent predictors (Table 5).

**Table 5:** Predictive power of parameters to distinguish between NMDCC subjects who developed symptomatic DCM (*n* = 15) and those that remained asymptomatic (*n* = 97): multivariate model based on step‐wise analysis of data

Parameter	Multivariate‐adjusted logistic regression models	
	Odds ratio (95% CI)	*p*
Radiculopathy	5.208 (1.288; 21.057)	**.021**
CR ≤ 4.0	5.613 (1.451; 21.708)	**.012**
CSA (mm^2^) ≤ 70.1	6.176 (1.608; 23.719)	**.008**

CR, compression ratio; CSA, cross‐sectional spinal cord area; EMG, electromyography; NMDCCC, nonmyelopathic degenerative cervical cord compression. Significant independent predictors are expressed in bold type.

## 4 DISCUSSION

This contribution reports the results of a validation study on the predictors for neurological dysfunction in the nonmyelopathic patient with degenerative cervical spinal cord compression. In a sample of subjects with NMDCC that included individuals with no signs and symptoms related to degeneration of the cervical spine, it emerged that cervical radiculopathy is the most important independent predictor for development of DCM. In addition, it established the independent predictive power of certain MRI parameters: CSA < 70.1 mm^2^ and CR < 0.4.

In a previous study, with a cohort of 199 NMDCCC individuals followed for 48 months, the authors documented the predictive value of cervical radiculopathy and electrophysiological signs of cervical cord dysfunction detected with SEP and MEP. This cohort, however, included nonmyelopathic but not completely asymptomatic cases, referred to a neurologist for radiculopathy or cervical pain. In this study, 37.5% of nonmyelopathic subjects had the least severe type of compression (type I) and 20.5% the most severe, type IIb. The data from the previous study (Bednařík et al., 1998) were re‐evaluated, and the proportions of types I and IIb proved different, with a lower proportion of type I (25.6%) and a higher proportion of type IIb (36.2%). Similarly, CSA < 70 mm^2^ was present in 22.3% of individuals in this study compared with 39.7% in the previous one. Thus, subjects in the former study were largely more severe, although still myelopathy‐free cases compared with this study, and this probably accounts for the partial discrepancy between the lists of independent predictors in the two studies and for why CSA and CR were disclosed as independent predictors for DCM development. These parameters have been shown to have high reliability in the assessment of cervical cord compression (Karpova et al., 2013; Kovalová, Bednařík, Keřkovský, Adamová, & Kadaňka, 2015). It is not surprising that adding completely asymptomatic subjects to our study group led to a lower proportion of NMDCCC individuals developing DCM in comparison with the former study (13.4% over 3 years and 7.3% during the first year in comparison with 22.6% over 48.4 months and 8.0 during the first year).

The main limitation of this study is the low number of outcome events in relation to the high number of potential predictors, which weakened the statistical evaluation. In contrast to radiculopathy, which proved a significant predictor in both the current and the previous study (Bednarik et al., 2008) and is generally accepted as such (Wilson et al., 2013), MRI parameters should be considered as preliminary predictors awaiting further confirmation.

Reliable detection of especially early stages of symptomatic DCM is a crucial point of the study. Although previously used diagnostic criteria for DCM were neither standardized nor consistent across published studies, recent and current studies have defined DCM by the presence of at least one neurological sign and at least one neurological symptom in addition to a positive MRI for compression of the cord (Amenta et al., 2014; Kalsi‐Ryan, Karamidas, & Fehlings, 2013).

Definition of MRI criteria for degenerative cervical cord compression is essential for reliable and reproducible diagnosis of DCM. In general, spinal cord compression can be described based on the appearance or by measuring a ratio between the anteroposterior diameter at the compressed site and that of a noncompressed site, a ratio between the anteroposterior diameter and the transverse diameter (i.e., CR), or CSA at the region of compression (Nouri, Martin, Mikulis, & Fehlings, 2016). MRI T1/T2 signal changes, although frequently detected in DCM, are neither sensitive nor specific for degenerative cervical cord compression and are invaluable to the diagnosis of DCM (Kalsi‐Ryan et al., 2013; Wilson et al., 2013). Regardless of the method used, the objective of especially quantitative measurements is to determine the severity of spinal cord compression rather than to detect especially subtle focal compressions.

The used MRI criterion for cervical cord compression based on subjective evaluation of a spinal cord contour or shape might be considered controversial. In our previous studies on that topic (Bednarik et al., 2004, 2008), we used the presence of impingement (i.e., focal change of contour) and/or CR < 0.4 as MRI criteria for cervical cord compression. However, using these criteria might have prevented less severe circular compressions from inclusion into the study and the compression ratio from showing off its predictive value.

We addressed the issue of an optimal quantitative imaging criterion for cervical cord compression in a recent cross‐sectional study of a large cohort of randomly recruited individuals (Kovalova et al., 2016). We used the same qualitative criterion (a change in spinal cord contour) as a gold standard and validated several quantitative MRI parameters for their sensitivity and specificity to discriminate between nonmyelopathic compression and no compression. An anteroposterior diameter of the cervical spinal canal of <9.9 mm was associated with the highest probability of MRI‐detected nonmyelopathic cervical cord compression in comparison with CR or CSA, which represent more severe circular compressions and are, on the contrary, more valuable in discrimination between nonmyelopathic compression and symptomatic DCM (Kovalova et al., 2016). We, thus, believe that the use of subjective evaluation of a change in the spinal cord contour or shape compared to that of the neighboring segment and based on agreement of two neuro‐radiologist is a legitimate criterion for definition of MRI signs of degenerative cervical cord compression in this study. Quantitative MRI parameters—CR and CSA—proved that especially more severe compressions increase the risk for development of symptomatic DCM and established cut‐offs might be used for stratification of a practical management of NMDCCC cases in addition to already known risk factors.

In NMDCCC cases with already detected MRI signs of cervical cord compression, progression into symptomatic myelopathy is based on clinical presentation. Symptoms, especially gait disturbance and loss of sensation, are the most commonly identified presenting symptoms (Kalsi‐Ryan et al., 2013), and our findings are similar. Myelopathic signs, although necessary for confirmation of myelopathic origin of otherwise unspecific symptoms, such as gait disturbance, are usually a hallmark of more advanced stage of myelopathy. Assessment tools to better define and document impairment and function quantitatively will be useful in identifying the actual clinical presentation and the impact on independence for these individuals (Kalsi‐Ryan et al., 2013). Quantified walk and run are definitely among those assessment tools. Gait or run impairment, however, can have quite a broad range of clinical presentations. We used quantified gait and run not for definition of symptomatic DCM, but as another possible predictor for progression of the disease. Prolonged gait or run proved to be able to discriminate/predict those patients with higher risk of developing symptomatic myelopathy. Lower statistical power of our study due to low number of outcome events in relation to the high number of potential predictors might be the reason why these functional tests, as well as some other predictors, did not prove to be an independent predictors using multivariate analysis. They are, however, promising and worthy further evaluation.

The degenerative compression is certainly a continuum with increased severity of compression and concomitant dysfunction/impairment of spinal cord. As it is not possible to differentiate reliably between symptomatic and nonmyelopathic cervical cord compression cases exclusively on clinical grounds, this limitation could lead to some confusion in terminology. One might speculate whether patients with MRI signs of cervical cord compression and abnormal conduction across spinal cord tracts proved by SEPs or MEP, those with MRI intramedullar signal changes, or those with prolonged time on quantified walk are really nonmyelopathic. Nevertheless, the current concept of symptomatic DCM is based on the presence of clear clinical symptoms and signs, and those “abnormal” or “subclinical” parameters increasing the risk for development of symptomatic myelopathy might define a subgroup of degenerative compressions that might be labeled as high‐risk NMDCCC or “presymptomatic myelopathy.”

In conclusion, previously and recently identified predictors of DCM development in NMDCCC individuals could help the decision‐making process for preventive surgical decompression and, more importantly, in defining a subgroup of NMDCCC individuals at higher risk of DCM, among whom a randomized trial evaluating the benefit of such decompression would be justifiable.

## CONFLICT OF INTEREST

The authors declare no conflict of interest.
